# Heart rate variability and perinatal depressive symptoms: A scoping review protocol

**DOI:** 10.1016/j.bbih.2024.100885

**Published:** 2024-10-05

**Authors:** Claudio Singh Solorzano, Marta Spinoni, Maria Grazia Di Benedetto, Alessandra Biaggi, Moira Marizzoni, Elena Gatti, Cristina Festari, Michela Pievani, Caterina Grano, Annamaria Cattaneo

**Affiliations:** a Laboratory of Alzheimer's Neuroimaging and Epidemiology, IRCCS Istituto Centro San Giovanni di Dio Fatebenefratelli, Brescia, Italy; b Department of Psychology, Sapienza University of Rome, Roma, Italy; c Biological Psychiatry Unit, IRCCS Istituto Centro San Giovanni di Dio Fatebenefratelli, Brescia, Italy; d Department of Pharmacological and Biomolecular Sciences, University of Milan, Milan, Italy

**Keywords:** Depression, Depressive symptoms, Heart rate variability, Pregnancy, Post-partum, Perinatal period

## Abstract

**Objective:**

An emerging marker of depression in the perinatal period is represented by a reduction in the autonomic nervous system (ANS) activity, reflected by heart rate variability (HRV). This scoping review aims to map the association between HRV and depression during the perinatal period and to understand its potential clinical implications.

**Introduction:**

Previous evidence associated ANS dysfunction and depressive symptomatology in the general population. Few observational and intervention studies investigated how HRV could be related to both pre- and post-partum depressive symptoms. However, high heterogeneity in the study designs and methods has been reported. Therefore, this scoping review plans to combine all these findings to build a starting point for future research.

**Inclusion criteria:**

This scoping review will consider articles focusing on the association between HRV and depression in the peripartum and – when available – on the impact of interventions on HRV and how this correlates with changes in depressive symptoms. Studies will be included with no restrictions on participants’ age, peripartum time points for the assessment, and HRV parameters collected.

**Methods:**

We will perform a systematic search using the Medline (PubMed), PsychInfo, and Web of Science (WoS) databases. Two authors will independently screen titles, abstracts, and then full-text articles that meet the inclusion criteria. The review will include only journal articles published in English, with no time limitations. Data will be extracted and presented in tables and/or graphical representations to summarise and describe the results. Extracted data will be reported in a comprehensive summary.

## Highlights

• In peripartum, heart rate variability (HRV) is associated with depression.
• Few intervention studies act on HRV to improve depressive symptoms.
• Studies investigating these topics have high levels of heterogeneity.
• The protocol set the stage for a scoping review of the topic.

## 1 Introduction

It is well known that depression ranks as the most widespread mental health disorder, significantly contributing to the overall global burden worldwide (GBD 2019 Mental Disorders Collaborators, 2022). Recent evidence shows that women are more likely to have depression compared to men (Kuehner, 2017). Beyond the social and educational factors, one key explanation could be related to the different functioning and regulation of biological systems between males and females (Di Benedetto et al., 2024). Indeed, literature data show that depression is strictly related to physiological processes, homeostatic changes, and hormonal fluctuations that are more common during women's development and life transitions (Kundakovic and Rocks, 2022).

Pregnancy is a physiological phase where psychological, social and biological changes can contribute to an increased vulnerability to developing depression (Halbreich, 2005). A recent meta-analysis on depression among perinatal women reported a prevalence of 28.5% for pre-partum depression and 27.6% for post-partum depression (Al-abri et al., 2023). Perinatal depressive symptoms do not generally appear to differ from general depressive symptoms. However, the rapid biological alterations during pregnancy and the presence of a newborn child in the post-partum are specific aspects that could affect depressive symptomatology. For instance, specific symptoms of perinatal depression may include mood lability, anxiety, irritability, feeling of inadequacy as a parent, pervasive concerns about the baby's health and feeding, feeling of being overwhelmed, and thoughts of harming themselves or the child (Stewart and Vigod, 2016, 2019). On the other side, somatic symptoms related to depression (e.g., sleep disruption, fatigue) overlap somatic complaints during pregnancy, making difficult the detection of depressive symptoms during the perinatal period (Nylen et al., 2013; Yonkers et al., 2009). Several studies investigated socio-demographic, obstetric, psychological, social and biological risk factors for perinatal depression, highlighting the crucial role of the change of biological systems functioning during the perinatal period in the pathophysiology of depression (Biaggi et al., 2016; Hutchens and Kearney, 2020; Payne and Maguire, 2019; Stewart and Vigod, 2016; Yim et al., 2015).

In this scoping review, we will focus on Heart Rate Variability (HRV) as a marker of depression in the perinatal period. HRV is an easy-to-collect parameter used to measure the Autonomic Nervous System (ANS) activity. It represents the fluctuation in the time intervals between heartbeats (Malik et al., 1996) and reflects the balance between the Sympathetic Nervous System (SNS) and Parasympathetic Nervous System (PNS) branches that innervate the heart (Shaffer et al., 2014). In rest conditions, a healthy heart rate fluctuates in a complex and flexible way to self-regulate and restore equilibrium whenever it gets disrupted (Shaffer and Ginsberg, 2017). In depression, previous evidence reported a reduction of resting parasympathetic activity as measured through vagally-mediated HRV (vmHRV) parameters (e.g., High-Frequency HRV, the root mean square of the successive differences between normal heartbeats – rMSSD, etc.) (Shir and Hanna, 2024; Wang et al., 2023). HRV is a modifiable physiological factor that can represent an attractive therapeutic target. For example, non-pharmacological interventions (i.e., respiration training or relaxation practices) have shown the potential to enhance psychological well-being (Goessl et al., 2017; Khazan, 2013).

The neurovisceral integration model (Thayer et al., 2009; Thayer and Lane, 2000) explains the association between HRV and depression, hypothesising that the neural network implicated in the self-regulation and adaptability of psychological processes (i.e., emotional and cognitive regulation) is also involved in the regulation of cardiac autonomic activity (Balzarotti et al., 2017; Forte et al., 2019). The association between HRV and emotional and cognitive regulation is related to the Central Autonomic Network (CAN) activity (Thayer et al., 2012), a circuit partially overlapping with the salience network that consists of cortico-limbic (e.g., anterior and mid-cingulate cortex, anterior insula, central nucleus of the amygdala, hypothalamus), and brainstem regions (e.g., periaqueductal grey matter, the nucleus of the solitary tract) (Ferraro et al., 2022; Mulcahy et al., 2019; Thayer et al., 2012). The CAN control of cardiac activity originates from the interaction among different neural structures in a feedback-based complex system that leads to ANS modulation that, in turn, affects heart activity (i.e., increase of heart rate (HR) and decrease of vmHRV). Literature shows that CAN activity also involves emotional and cognitive regulation, especially the prefrontal cortex and amygdala (Park and Thayer, 2014; Thayer et al., 2012). The activity of these brain regions modulates human behaviours by the interconnection between higher-level psychological functions and autonomic regulation of the heart. The CAN integrates the internal and external input, assesses the threat and safety of each situation, and flexibly adjusts emotional and executive functions (Seeley, 2019). The output of these neural activities affects the heart through the ANS. Therefore, HRV – especially vmHRV – reflects the functional capacity of CAN brain regions that support cognitive and emotional self-regulation (Shaffer et al., 2014; Thayer et al., 2009). According to this model, the nonadaptive and prolonged inhibition of prefrontal cortex activity and the related effect on the amygdala can lead to a disruption of adaptive and goal-directed behaviours that, in turn, could lead to perseverative negative thoughts, hypervigilance, emotional instability, and then to psychopathology (Park et al., 2012; Thayer et al., 2012). Specifically, pharmacological and neuroimaging-based studies showed that hypoactivity of the prefrontal cortex is associated with an increase in HR and a decrease in vmHRV, supporting the view of an association between lower HRV vagal parameters and a higher risk of mental disorders (Thayer et al., 2009; Thayer and Lane, 2009).

During pregnancy, the ANS activity undergoes wide changes to maintain the body's health homeostasis and adapt to the growing fetus's physiological needs (Brooks et al., 2020). Several studies indicated a physiological increase in the basal heart rate, a predominance of SNS, and a reduction of PNS activity – reflected by the reduction of vmHRV parameters – during the second and third trimester of pregnancy, with a normalisation of the pre-pregnancy ANS activity starting from the first days before childbirth and during the post-partum period (Brown et al., 2021; Fu, 2018; Rowan et al., 2022). Moreover, literature reported that primiparous women have lower levels of mvHRV compared to multiparous women (Solanki et al., 2020) and also that health habits during pregnancy (e.g., sleep quality, physical activity) could influence perinatal ANS activity (May et al., 2016; Shiga et al., 2012). All these physiological variations are mainly outlined by anatomical, hormonal, and immunological adaptive changes during normal pregnancy (Abu-Raya et al., 2020; Brooks et al., 2020; Kazma et al., 2020). On the other side, dysfunctional and nonadaptive alterations of perinatal PNS could be associated with emotional and psychological disorders (Kimmel et al., 2021) Few studies have investigated the association between vmHRV and perinatal depressive symptomatology (Eriksson et al., 2024; Shah et al., 2020; Shea et al., 2008; Singh Solorzano et al., 2022). For instance, Shea et al. (2008) examined the association between depression and ANS function during the third trimester of pregnancy by comparing HRV parameters obtained from a 24-h ECG in depressed and healthy pregnant women. Their findings showed that depressed pregnant women have lower values of vmHRV than the control group. Another cross-sectional study reported an imbalance between SNS and PNS and lower levels of baroreflex sensitivity associated with depressive symptoms during pregnancy (i.e., between 12 and 30 weeks) (Shah et al., 2020). On the other hand, Kimmel et al. (2021) found no association between different HRV parameters and the presence of a major depressive disorder in pregnant women during the third trimester of pregnancy. A few intervention studies corroborate these findings, showing that HRV-biofeedback (Beckham et al., 2013; Kudo et al., 2014) or mindfulness-based treatments (Braeken et al., 2017; Rådmark et al., 2023) might be promising interventions to reduce depressive symptoms during the perinatal period.

However, both observational and intervention studies showed high heterogeneity in the assessment of perinatal depression (e.g., different gestational periods and tools to assess depressive symptoms) and HRV (e.g., different parameters and collection methods), limiting the interpretation of the results and their potential clinical implications. Therefore, we will conduct a scoping review to summarise the main findings on the association between perinatal HRV and depressive symptoms. Moreover, we will also consider evidence on HRV modulation-based interventions (e.g., HRV-biofeedback) in the context of perinatal depression. A preliminary search of Scopus, MEDLINE, and the Cochrane Database of Systematic Reviews was conducted on the 22nd of July 2024. No current or ongoing scoping reviews and systematic reviews on the same subject were identified in this preliminary search. The protocol was written according to the Joanna Briggs Institute (JBI) protocol scoping review template (Peters et al., 2024).

## 2 Review question

(a) What is the cross-sectional association between HRV and depressive symptoms during the perinatal period?
(b) What is the longitudinal association between HRV and depressive symptoms during the perinatal period?
(c) Which interventions act on HRV and show a beneficial effect on depressive symptoms during the perinatal period?

## 3 Eligibility criteria

Eligibility criteria were established in line with JBI scoping review guidance (Peters et al., 2024) by referring to the population, concept, context, and type of sources framework. We sum up the inclusion and exclusion criteria in Table 1.

**Table 1:** Inclusion and exclusion criteria for the considered studies.

Inclusion criteria	
Population	o Any participant's age, pregnancy trimester or post-partum time point, and past psychiatric condition. o Current presence of depressive symptoms or major depressive disorder.
Concept	o Depressive symptoms: depressive symptoms or major depressive disorders at any peripartum time point. oHRV: any parameter derived from ECG or other validated devices. oParasympathetic-based intervention: any intervention studied aiming to improve depressive symptomatology and with the measure of any HRV parameter.
Context	o Any socio-economic context and geographical location.
Type of sources	o Quantitative research studies: analytical observational and interventional studies.
**Exclusion criteria**	
o Participants with a diagnosis of other psychiatric disorders.	
o Studies with HRV parameters derived from not-validated photoplethysmographic devices.	
o Non-English studies.	
o Qualitative and mixed methods research studies, grey literature, conference abstracts, and thesis/dissertations.	

## 4 Population

The population will be represented by women during the peripartum period with no limitations for inclusion criteria on age, the gestational trimester of pregnancy or the post-partum period, and past psychiatric conditions. If literature data allows, we will focus our discussion on specific subgroups of pregnancy trimester or post-partum periods. Women with a pre-pregnancy or current presence of depressive symptoms or major depressive disorder (MDD) will be included. Diagnosis of other psychiatric disorders will represent an exclusion criterion, considering that different patterns of association between mental conditions different from depression and HRV parameters could exist (Eriksson et al., 2024; Kimmel et al., 2021).

## 5 Concept

The concepts to be explored in the present scoping review include pre-partum depressive symptoms, post-partum depressive symptoms, HRV, and parasympathetic-based intervention. In the included studies, depressive symptoms could be present in pre-pregnancy and have to be measured during pregnancy or in the post-partum period, with no time limits. This lack of time constraints derived from research and clinical practice evidence reporting the potential onset of depressive symptoms beyond 12 months after childbirth (Putnam et al., 2015; Stewart and Vigod, 2016; Wang et al., 2021). Therefore, we will describe the results for the perinatal period (i.e., from pregnancy to the first year post-partum) (O'Hara and Wisner, 2014) and the results that detect depressive symptoms beyond this range. The choice is related to maintaining the broadest possible literature research, according to the explorative nature of the review.

As one of the main aims of this scoping review is to shed light on the cardiac sympathetic and parasympathetic activity changes in peripartum depression, we will include all studies that assess the most common HRV parameters (Shaffer and Ginsberg, 2017), derived from both electrocardiographic (ECG) or photoplethysmographic (PPG) signal. Considering ECG as the gold standard for measuring cardiac signals, we will include studies with PPG assessment of cardiac signals only if the methods were validated in previous studies.

We will also include intervention studies aiming at improving parasympathetic activity to reduce depressive symptomatology in the peripartum, with no exclusion criteria for the type of mind-body treatment (e.g., HRV biofeedback, mindfulness, etc) (Oyarzabal et al., 2021). The review will include intervention studies in which depressive symptomatology and HRV parameters were assessed before and/or after the intervention. Any theoretical or methodological limit in the included interventional studies will be discussed.

## 6 Context

This review will consider studies conducted in any socio-economic context and geographical location.

## 7 Type of sources

This scoping review will mainly consider peer-reviewed quantitative research studies. In particular, we will include analytical observational studies (e.g., prospective and retrospective cohort studies, case-control studies, analytical cross-sectional studies) and interventional studies (e.g., randomised controlled trials, non-randomised controlled trials, pre-post studies). In addition, systematic reviews that meet the inclusion criteria will also be considered, depending on their research questions. We will exclude qualitative and mixed methods research studies. Grey literature, conference abstracts, and thesis/dissertations will not be considered for inclusion in this scoping review.

## 8 Methods

The present scoping review will follow the published JBI methodology (Peters et al., 2020, 2024) and will be conducted in accordance with the PRISMA-ScR (Preferred Reporting Items for Systematic Reviews and Meta-Analyses Extension for Scoping Reviews) (Tricco et al., 2018). The title, objectives, inclusion criteria, and general methods for this scoping review have been included in Open Sciences Framework (OSF) registries (https://doi.org/10.17605/OSF.IO/DPA8K).

## 9 Search strategy

Studies published in English, with no time delimitation, will be included. The databases to be searched will include MEDLINE (PubMed), PsychInfo, and Web of Science. A three-step search strategy will be applied (Peters et al., 2024). In particular, the three steps are detailed below.

Step 1: A first preliminary MEDLINE (PubMed) and PsychInfo search will be performed to identify articles focused on the topic of interest. The text words contained in the titles and abstracts of selected articles and the index terms used to describe the articles will be used to develop step 2.
Step 2: A second search using the identified keywords and index terms will be conducted across all the included databases. An example search strategy for MEDLINE (PubMed) is included in Appendix A. This consists of three main parts joined by Boolean operators: the first restricts the search to studies on the peripartum period, the second includes the concept of depression, and the third focuses on HRV (using all synonymous and related terms we know are relevant). Moreover, to answer the third question of the review, a last filter will be used to consider intervention studies. Ultimately, our strategy will consider synonyms, related terms, Boolean operators, and explored Medical Subject Headings (MeSH) terms. The search strategy, including all identified keywords and index terms, will be adapted for each included database.
Step 3: The reference lists of all the articles included in the review will be screened for additional papers.

### 9.1 Study/sources of evidence selection

Following the search, all the identified citations will be collated and uploaded to EndNote™ X8 software, and duplicates will be removed. Titles and abstracts will be imported into Rayyan (Ouzzani et al., 2016) and screened by two independent reviewers (CSS and MS) for assessment against the inclusion criteria defined for the scoping review. Studies that meet or could potentially meet the inclusion criteria will be retrieved in full, and their details will be imported into Rayyan and assessed against the inclusion criteria. Full-text studies that do not meet the inclusion criteria will be excluded, and the final scoping review report will provide reasons for exclusion. The search results will be reported in full in the final report and presented in a PRISMA flow diagram. Any reviewer disagreements will be resolved through discussion or with an additional expert reviewer (CG).

### 9.2 Data extraction

Two independent reviewers (CSS and MS) will extract data from the selected papers using a data extraction tool developed by the reviewers based on the JBI template (Peters et al., 2024). A draft data extraction form is presented in Appendix B. The data extraction tool will be revised as necessary during the data extraction process, with modifications detailed in the scoping review. Any reviewer disagreements will be resolved through discussion or with an additional expert reviewer (CG).

### 9.3 Quality assessment

Despite scoping reviews do not require the use of a standardised quality assessment of included studies as systematic reviews (Arksey and O’Malley, 2005; Peters et al., 2024; Tricco et al., 2018), the debate is still open (Pham et al., 2014). We will assess the quality of the studies by analysing the internal and external validity of the included studies (Petticrew and Roberts, 2008). Using the JBI Critical Appraisal Checklist tools (Moola et al., 2024), we will evaluate the sampling method, data collection method and instruments, the context of the study and whether the results and conclusions were transparent. Each checklist component was rated as yes, no, unclear, or not applicable. Two authors (CSS and MS) independently evaluated the quality of each, and disagreements were resolved by discussion with an additional expert reviewer (CG).

### 9.4 Data analysis and data presentation

Data extracted from each included article will be organised and/or presented in diagrammatic format. Tables and/or diagrams will be supplemented by a narrative text to describe the associations between peripartum HRV and depressive symptomatology and – if available – evidence of the effect of HRV-related interventions on depressive symptoms. The results of the quality assessment were reported in a table. The review will identify research gaps in the existing literature, showing available results and how they relate to clinical practice and future research.

## Funding

The IRCCS Fatebenefratelli is partially supported by the Italian Ministry of Health (Ricerca Corrente).

## Statement

During the preparation of this work the author(s) did not use generative AI and AI-assisted technologies.

## CRediT authorship contribution statement

**Claudio Singh Solorzano:** Writing – review & editing, Writing – original draft, Visualization, Resources, Project administration, Methodology, Investigation, Data curation, Conceptualization. **Marta Spinoni:** Writing – review & editing, Resources, Methodology, Investigation, Data curation. **Maria Grazia Di Benedetto:** Writing – review & editing, Resources. **Alessandra Biaggi:** Writing – review & editing, Resources. **Moira Marizzoni:** Writing – review & editing, Resources. **Elena Gatti:** Writing – review & editing, Methodology. **Cristina Festari:** Writing – review & editing, Methodology. **Michela Pievani:** Writing – review & editing, Supervision. **Caterina Grano:** Writing – review & editing, Supervision. **Annamaria Cattaneo:** Writing – review & editing, Supervision, Resources, Project administration, Conceptualization.

## Declaration of competing interest

The authors declare that they have no known competing financial interests or personal relationships that could have appeared to influence the work reported in this paper.

## Figures and Tables

**Table:** 

Search	Query	Records
#1 Peripartum	"pregnancy"[Mesh] OR "pregnant women"[Mesh] OR "postpartum period"[Mesh] OR "gravidity"[Mesh] OR "peripartum period"[Mesh] OR "maternal"[tw] OR "mother"[tw] OR "postnatal"[tw] OR "prenatal"[tw] OR "antenatal"[tw] OR "prepartum"[tw] OR "pre-partum"[tw] OR "post-partum"[tw] OR "pre-natal"[tw] OR "post-natal"[tw] OR "ante-natal"[tw]	1,406,431
#2 Depression	"depression"[Mesh] OR "depressive disorder"[Mesh] OR "depress∗" [tw] OR "affective"[tw] OR "mood"[tw]	794,782
#3 HRV	"Heart Rate Variability"[tw] OR "HRV"[tw] OR "sympathetic"[tw] OR "parasympathetic"[tw] OR “autonomic”[tw] OR "vagal"[tw] OR "vagus"[tw] OR "SDNN"[tw] OR "SDRR"[tw] OR "SDANN"[tw] OR "pNN50"[tw] OR "RMSSD"[tw] OR "TINN"[tw] OR "ULF"[tw] OR "VLF"[tw] OR "LF"[tw] OR "HF"[tw] OR “LFnu”[tw] OR “HFnu”[tw] OR "SD1"[tw] OR "SD2"[tw] OR "ApEn"[tw] OR "SampEn"[tw] OR "DFA"[tw]	317,429
#4	#1 AND #2 AND #3	489
**Sub-search on treatment**		
#5	"therapeutics"[MeSH] OR "treatment outcome"[MeSH] OR "intervention∗" [tw] OR "treatment"[tw] OR "therap∗"[tw] OR "trial∗"[tw] OR "randomized controlled”[tw] OR "pilot"[tw]	13,567,622
#6	#4 AND #5	199

**Table:** 

**Scoping Review Details**
Scoping Review title:
Review objective/s
Review question/s:
**Article details**
Authors
Publication year
Study design
Aim and/hypotheses
Outcome measures
Key findings
**Population characteristics**
Sample size
Age
Pre-pregnancy BMI
Pregnancy BMI
Pregnancy trimester (if applicable)
Post-partum time point (if applicable)
Parity
Medical conditions (e.g., hypertensive disorders, gestational diabetes, preeclampsia, etc.)
Current or past history of depressive symptoms or major depressive disorder
Current or past pregnancy adverse events (e.g., preterm delivery, miscarriage, etc.)
**Concept**
Questionnaire/s used to measure depressive symptoms.
Method of collection of HRV
Collected parameters of HRV
Type of intervention (if applicable)
**Other**
Author-identified limitations
Author-identified research opportunities

## Data Availability

No data was used for the research described in the article.

## Appendix A Search strategy

Medline (PubMed).

Search conducted: 22nd of July 2024.
